# Polymyxins and Their Potential Next Generation as Therapeutic Antibiotics

**DOI:** 10.3389/fmicb.2019.01689

**Published:** 2019-07-25

**Authors:** Martti Vaara

**Affiliations:** ^1^Northern Antibiotics Ltd., Espoo, Finland; ^2^Department of Bacteriology and Immunology, Helsinki University Medical School, Helsinki, Finland

**Keywords:** polymyxin B, colistin, extremely resistant (XDR), Gram-negative bacteria, improved polymyxins

## Abstract

The discovery of polymyxins, highly basic lipodecapeptides, was published independently by three laboratories in 1947. Their clinical use, however, was abandoned in the sixties because of nephrotoxicity and because better-tolerated drugs belonging to other antibiotic classes were discovered. Now polymyxins have resurged as the last-resort drugs against extremely multi-resistant strains, even though their nephrotoxicity forces clinicians to administer them at doses that are lower than those required for optimal efficacy. As their therapeutic windows are very narrow, the use of polymyxins has received lots of justified criticism. To address this criticism, consensus guidelines for the optimal use of polymyxins have just been published. Quite obviously, too, improved polymyxins with increased efficacy and lowered nephrotoxicity would be more than welcome. Over the last few years, more than USD 40 million of public money has been used in programs that aim at the design of novel polymyxin derivatives. This perspective article points out that polymyxins do have potential for further development and that the novel derivatives already now at hand might offer major advantages over the old polymyxins.

## Introduction: the Old Polymyxins

In 2010, the World Health Organization (WHO) stated that antibiotic resistance is one of the three greatest threats to human health. Many strains of Gram-negative bacteria have now developed resistance to practically all currently used antibacterial agents, including carbapenems. These strains are spreading globally and this narrows down the therapeutic options (Theuretzbacher, 2017; Wright et al., 2017; Bonomo et al., 2018; Tumbrarello et al., 2018; Cox et al., 2019; Koulenti et al., 2019). At the same time the few agents that remain potentially usable do have their defects, too. Low serum and urine levels shadow the use tigecycline (Tumbrarello et al., 2018). Ceftazidime–avibactam (CZA), imipenem–cilastatin–relebactam (I–R), and meropenem–vaborbactam (MVB) are not effective against strains that produce carbapenemases belonging to the metallo-beta-lactamase group, i.e., to Class B beta-lactamases (Wright et al., 2017; Tumbrarello et al., 2018; Koulenti et al., 2019). Furthermore, Class A carbapenemase-producing KPC strains that are mutationally resistant to CZA are emerging, and resistance is observed following relatively short courses of therapy (Shields et al., 2017; Wright et al., 2017; Tumbrarello et al., 2018). CZA is the eldest of the novel beta-lactam/beta-lactamase inhibitor combinations, and the question arises, whether a similar resistance development will be seen with the newer beta-lactam–beta-lactamase inhibitor combinations such as I–R, MVB, and the related derivatives (Wright et al., 2017). The neoaminoglycoside plazomicin entered the clinics in 2018 but it is not active against strains in which the target is altered due to 16S rRNA methylation (Wright et al., 2017; Theuretzbacher and Paul, 2018; Koulenti et al., 2019). Future does not look bright, as today only a few novel antibiotics against the extremely drug-resistant Gram-negative bacteria are under development (WHO, 2019).

**FIGURE 1:**
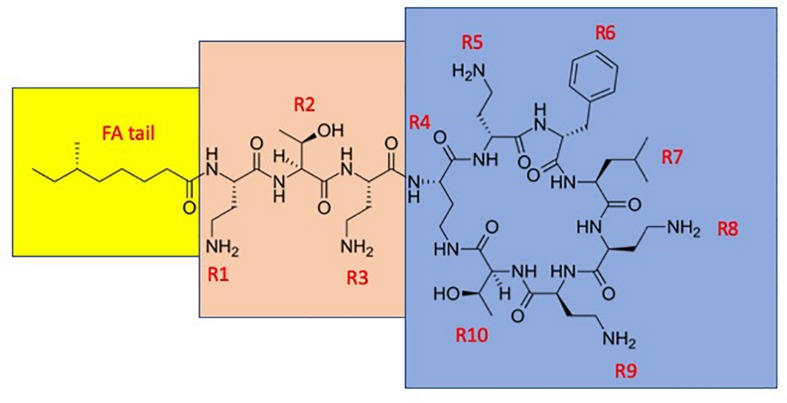
The structure of polymyxin B1. The fatty acyl tail is highlighted with yellow, the linear “panhandle” part (i.e., the residues R1–R3) with pink, and the cyclic heptapeptide part (i.e., residues R4–R10) with blue.

Polymyxins are cyclic lipodecapeptide antibiotics (Figure 1 and Table 1). Out of this group, polymyxin B (PMB) and colistin (administered as an antibacterially inactive prodrug colistin methanesulfonate, also known as colistimethate, CMS) are in current clinical use. They are highly basic due to their five free amino groups and quite effective against Gram-negative bacteria, such as the majority of Enterobacteriaceae, as well as *Acinetobacter baumannii* and *Pseudomonas aeruginosa*. Polymyxins act specifically on Gram-negative bacteria and are rapidly bactericidal, while Gram-positive bacteria, eukaryotic microbes, and mammalian cells are typically unaffected (HsuChen and Feingold, 1973). The antibacterial potencies of PMB and colistin are identical (Gales et al., 2011; Sader et al., 2015). Polymyxins first interact with the anionic lipopolysaccharide (LPS) molecules, exclusively present in Gram-negative bacteria and located in the outer leaflet of their outer membrane (OM), as reviewed by Vaara (1992). The lipid A part of LPS carries anionic phosphate and pyrophosphate groups, and the cationic polymyxins bind into these groups (Vaara, 1992). This interaction results in the release of LPS and the disorganization, restructuring, and generation of cracks in the OM, leading to loss of function as a permeability barrier. In electron micrographs, polymyxins cause blebbing of the OM vesicles outside the cell as well as typical “fingerlike projections” in the OM (Vaara, 1992). Furthermore, polymyxins cause leakage of the periplasmic contents, such as beta-lactamases, and make the OM permeable the many noxious agents such as antibiotics and lysozyme (Vaara, 1981, 1992). The final and lethal action of polymyxins is the damage to the cytoplasmic membrane. They cause leakage of cytoplasmic contents such as ATP (Murray et al., 2016). Additional possible mechanisms include inhibition of NADH-quinone oxidoreductase activity (Deris et al., 2014) as well as production of hydroxyl radicals (Sampson et al., 2012) and reactive oxygen species (Imlay, 2013).

**TABLE 1:** The structures of polymyxin B, colistin, and the novel polymyxin derivatives that display improved efficacy in animal infection models (compounds 4–10)^a,b^.

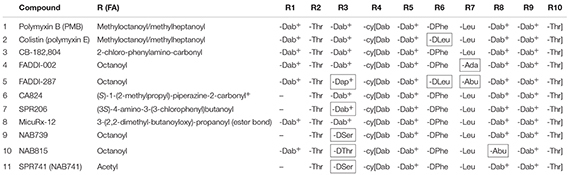

*The structure of one discontinued derivative (compound 3) as well as that of the potentiator compound SPR741 (NAB741) are also shown. ^*a*^Amino acyl residues that differ from those in polymyxin B are boxed. ^*b*^Abu, aminobutyryl; Ada, aminodecanoyl; Dap, diaminopropionyl; cy, cyclic portion indicated with brackets; Dab, diaminobutyryl; FA, fatty acyl.*

The clinical use of polymyxins was abandoned in the sixties because of nephrotoxicity and because better-tolerated drugs belonging to other antibiotic classes were discovered. Now, however, polymyxins have resurged as the last-resort drugs against extremely multi-resistant strains, even though their nephrotoxicity forces clinicians to administer them at doses that are lower than those required for optimal efficacy. The currently used polymyxins are drugs with very narrow therapeutic windows (Nation et al., 2019). Very recently, consensus guidelines for the optimal use of the polymyxins have been published (Tsuji et al., 2019). Recommendations were given on many important issues such as the dosing of polymyxins. In the therapy of invasive infections, PMB appears to be less nephrotoxic than CMS and hence preferred (Nation et al., 2019; Tsuji et al., 2019). Furthermore, PMB seems to have superior pharmacokinetic characteristics for infections in which it is important to achieve the desired concentration in plasma rapidly and reliably and to maintain it as well (Tsuji et al., 2019). In the therapy of lower urinary tract infections (i.e., bladder infections, cystitis), the bacteriologically inactive prodrug CMS is preferred, because it is effectively excreted into the bladder and subsequently hydrolyzed there to the bacteriologically active form, i.e., colistin (Tsuji et al., 2019). However, its pharmacokinetics, including the hydrolysis to active drug, shows substantial interpatient variability and this makes the choice of the proper dose difficult (Tsuji et al., 2019).

Polymyxin B and colistin are effectively bound to the brush-border membrane (BBM) of the proximal tubular kidney cells and taken up (reabsorbed) by these cells (Moestrup et al., 1995). At the molecular level, the binding and uptake takes place via the megalin-mediated endocytosis (Moestrup et al., 1995; Suzuki et al., 2013; Abdelraouf et al., 2014) but very recent studies indicate that human peptide transporter 2 (PEPT2) and carnitine/organic cation transporter 2 (OCTN2) are involved as well (Lu et al., 2016; Visentin et al., 2017). Once taken up by the cells, polymyxins inhibit the mitochondrial electron transport, i.e., respiration (as reviewed by Gai et al., 2019), cause increased superoxide production (Nation et al., 2019), and activate caspases that lead to cellular apoptosis (Visentin et al., 2017). Polymyxins also upregulate the cholesterol biosynthesis in the kidney cells (Weber et al., 2018). Whether this is an attempt to protect cells remains to be elucidated. Regarding protection, there have been many attempts to reduce the nephrotoxicity of polymyxins by adding simultaneously antioxidants (Gai et al., 2019; Nation et al., 2019). According to the consensus paper, the current data are insufficient to recommend routine administration of ascorbic acid or any other antioxidant for the prevention of nephrotoxicity (Tsuji et al., 2019).

## Improved Polymyxins

Even though the value of the polymyxins now used in the clinics is acknowledged, novel derivatives that are more effective and less toxic would be more than welcome. It should be noted that most of the antibiotics of the “golden age of antibiotic discovery” have now been replaced by their improved versions. That has happened to the archetypical beta-lactam antibiotic penicillin G; the first cephalosporin, cephalothin; the first tetracycline, chlortetracycline; the first aminoglycoside antibiotic streptomycin; and the first macrolide antibiotic erythromycin. The first clinically used antibacterial quinolone, nalidixic acid, was followed by a wide variety of better quinolones and fluoroquinolones. However, some of the antibiotic classes discovered decades ago have not yet been developed any further but are still in current use. These ancient “dinosaurs” of the antibiotic world include polymyxins.

One case has perhaps shadowed the development of improved polymyxins. The development of Cubist’s CB-182 804 (Table 1) was discontinued in 2010 after the Clinical Phase 1, apparently because of toxicity issues. It differed from PMB only by carrying a different fatty acyl group linked to the peptide part. Later on, Pfizer published their discovery that replacement of diaminobutyric acid (Dab) at the position R3 in the polymyxin peptide with diaminopropionic acid (Dap) (Table 1) reduced the cytotoxicity to kidney cell cultures and rat kidneys (Magee et al., 2013). However, this difference did not translate to dogs (Magee et al., 2013), and Pfizer dropped the program. Astra-Zeneca has also discontinued their polymyxin program (chemical structures not revealed).

On the other hand, there are others who do believe that polymyxins have potential for further development and recently many attempts have been made to design more effective and better tolerated derivatives. This work has been portrayed in extensive reviews (Vaara, 2013, 2018; Brown and Dawson, 2017; Rabanal and Cajal, 2017; Gallardo-Godoy et al., 2019).

Northern Antibiotics (NAB) made the strategic decision to develop less nephrotoxic derivatives that carry three positive charges only, in contrast to five in the old polymyxins. They showed that binding of PMB to kidney BBM (see above) does correlate with the number of positive charges in the molecule (Vaara et al., 2008; Shapiro, 2013). NAB739 and NAB815 (Table 1) are less nephrotoxic than PMB in cynomolgus monkeys (Vaara, 2018). Furthermore and quite importantly, both compounds are thus far the only polymyxins effectively excreted into urine (Vaara, 2018).

FADDI-02, FADDI-287, CA824, MicuRx-12, and SPR206 [formerly CA1206] (for the structures of these compounds, see Table 1) are superior to the old polymyxins in the rodent lung infection model with *A. baumannii* and/or *P. aeruginosa* (Velkov et al., 2014; Boakes et al., 2015; Gordeev et al., 2016; Brown and Dawson, 2017; Grosser et al., 2018b). In *Escherichia coli* systemic infection in mice, MicuRx-12 is slightly more effective than PMB (Gordeev et al., 2016). In mouse urinary tract *E. coli* infection, SPR206 displays activity similar or slightly superior compared to PMB (Grosser et al., 2018a) while the effective dose of NAB739 and NAB815 is only one-tenth of that of PMB (Vaara et al., 2018).

SPR206 (now in Clinical Phase 1) has an altered fatty acyl tail and a shortened linear part and appears to be less nephrotoxic than PMB in cynomolgus monkeys (Lister et al., 2018). Regarding several other derivatives, more results in non-human primates are awaited as the lowered nephrotoxicity has thus far been demonstrated only in rodents for the FADDI compounds (Sabet et al., 2016) which have altered amino acyl groups in the cyclic and linear parts, and for MicuRx-12 (Gordeev et al., 2016) in which the fatty acyl tail is linked to the cyclic part via an ester bond. In plasma, this ester bond enables the decomposition of the compound to a tail-less and apparently less toxic metabolite. Whether therapeutically effective amounts of the intact compound reach the infection focus before decomposition in indications such as complicated urinary tract infection (cUTI) remains to be evaluated. The ester bond compound MRX-8 is now reported to be in investigational new drug (IND)-enabling studies (Gordeev, 2018).

The U.S. National Institute of Allergy and Infectious Diseases (NIAID) has since 2012 funded the Monash University (Melbourne, Australia) by more than USD 25 million in programs that aim at the discovery of new polymyxins and the re-development of the old ones. This funding continues. The group of Prof. Roger Nation and Prof. Jian Li at the Monash University is today the world leader in polymyxin studies. In addition, NIAID has supported the preclinical studies on SPR206 by USD 6.3 million. Furthermore, the transatlantic funding instrument CARB-X that aims at accelerating global antibacterial innovation has recently granted MicuRx USD 5.2 million to be used in further development of their polymyxin derivatives.

Other examples attesting the hopes put on polymyxins include SPR741 (formerly NAB741; Vaara, 2019; Table 1) which passed successfully Clinical Phase 1 in late 2017. In contrast to other derivatives under development, SPR741 lacks the direct antibacterial activity, i.e., it is not bactericidal. However, it does sensitize the targets to more than 10 different partner antibiotics, normally active against Gram-positive bacteria only. Furthermore, it is synergistic with several beta-lactam antibiotics that are effective against Gram negatives, too. It is an excellent model for polymyxins that damage the outermost cell surface barrier, the OM and permeabilize it to other antibiotics. In 2017, CARB-X granted Spero Therapeutics USD 5.4 million for further studies on SPR741.

As a great number of research groups are currently developing novel polymyxin derivatives, one crucial practical aspect is worth noticing. The *in vitro* susceptibility studies in which novel polymyxin derivatives under development are compared to the old polymyxins and to each other are more demanding than many other susceptibility studies. The activity of polymyxins varies greatly depending on the test conditions used, including the choice of the growth medium. Furthermore, polymyxins are in a very significant degree adsorbed to (i.e., inactivated by) polystyrene microwell plates and other laboratory plastics in a time-dependent fashion (Karvanen et al., 2017; Poirel et al., 2017; Gallardo-Godoy et al., 2019). To enable proper comparison of the results, all research laboratories that develop novel polymyxins should use the CLSI/EUCAST standard that is currently used in the clinical laboratories in the determination of susceptibility to the old polymyxins. Accordingly, in the broth microdilution (BMD) method, cation-adjusted Mueller–Hinton broth (CA-MHB) should be used. To minimize the effect of adsorption, the bacterial inoculum should be administered without any delay to the wells containing polymyxins. Different derivatives may be adsorbed differently to the plastics, depending on their hydrophobicity, and perhaps on their cationic charge. The best way to eliminate errors caused by the adsorption is to add the compounds to the wells that already have the target bacteria in the growth medium. The other CLSI-approved reference method, agar dilution (AD) method, is not influenced by the time-dependent inactivation of polymyxins and hence may offer advantages over the BMD method. Even though more laborious than the BMD method, it is currently used in several laboratories (Vaara et al., 2008, 2017; Otter et al., 2017; Tyrrell et al., 2019).

## But What About the Acquired Polymyxin Resistance?

The recent appearance of mobile colistin resistance (*mcr*) genes has raised concern and a lot of publicity (Liu et al., 2016; Poirel et al., 2017). This type of polymyxin resistance (usually low-level resistance) may have its origin in China, where colistin was used as a feed additive in animal husbandry. Recently, China has announced to ban this use. Even more worrisome are the strains resistant to polymyxins by other mechanisms, such as those with altered *mgrB* that display high-level resistance (Poirel et al., 2017). In the recent SENTRY study (isolates collected worldwide in 2017), the frequency of colistin resistance was still very rare, in *Klebsiella pneumoniae* (*n* = 3753) only 0.4% and in *E. coli* (*n* = 7397) only 0.3% (SENTRY, 2019). However, among the global isolates of carbapenemase-producing *K. pneumoniae* (*n* = 1703) and *E. coli* (*n* = 407) collected during the SMART program in 2015–2016, the prevalence of colistin resistance was 21 and 14%, respectively (Lob et al., 2017). In 2015, the European Antimicrobial Surveillance Network (EARS-Net) registered 33,100 deaths due to infections caused by antibiotic-resistant bacteria. Out of these, 6.8% were caused by colistin-resistant *E. coli* or *K. pneumoniae* and 0.5% by colistin-resistant *A. baumannii* or *P. aeruginosa* (Cassini et al., 2019). Regarding *A. baumannii*, the international SENTRY program (1997–2016) reported that from the strains collected in Europe (*n* = 3275), 6.1% were polymyxin-resistant and 64.4% were meropenem-resistant (Gales et al., 2019). In the United States, the corresponding frequencies were 4.6 and 40.6% (*n* = 2461). Accordingly, polymyxin-resistant strains are still rather rare and active at least *in vitro* against the carbapenem-resistant strains of *A. baumannii.* However, the resistance may develop during the therapy (Tsuji et al., 2019).

As is the case for all antibiotics, polymyxin resistance will eventually become more common. In this respect, however, polymyxins seem to differ from all other antibiotics in a positive way. While not able to confer a lethal effect on the polymyxin-resistant strains, polymyxins and their novel derivatives seem to be able to sensitize polymyxin-resistant strains to various “partner antibiotics” or “anti-Gram-positive antibiotics” that enter the Gram-negative cell only if its OM permeability barrier is first damaged by polymyxins to make it more permeable to other noxious agents. Recent studies indicate that polymyxins sensitize polymyxin-resistant strains to such partner antibiotics, including rifampicin, azithromycin, clarithromycin, minocycline, fusidic acid, zidovudine, and several other drugs (Tascini et al., 2013; Brennan-Krohn et al., 2018; MacNair et al., 2018; Loose et al., 2019). The derivative NAB739 shares this property as well (Tyrrell et al., 2019). There are also attempts to assess whether polymyxins could be derivatized to cover strains resistant to the old polymyxins (Vaara, 2013; Brown and Dawson, 2017; Rabanal and Cajal, 2017; Gallardo-Godoy et al., 2019).

## Concluding Remark

Future will tell whether polymyxins join the expanding group of antibiotics developed from primitive ancestors to modern drugs. Improved polymyxins now under development respond to previous challenges and some of them may turn out to be more successful than the old polymyxins, thus strengthening our armature before the antibiotic resistance crisis that we face now grows into a catastrophe.

## Data Availability

All datasets analyzed for this study are included in the manuscript and the supplementary files.

## Author Contributions

The author confirms being the sole contributor of this work and has approved it for publication.

## Conflict of Interest Statement

MV is the co-founder, shareholder, and CEO of Northern Antibiotics Ltd., and a member of the Scientific Advisory Board of Spero Therapeutics.
