# Corrigendum: Polymyxins and Their Potential Next Generation as Therapeutic Antibiotics

**DOI:** 10.3389/fmicb.2019.02275

**Published:** 2019-10-01

**Authors:** Martti Vaara

**Affiliations:** ^1^Northern Antibiotics Ltd., Espoo, Finland; ^2^Department of Bacteriology and Immunology, Helsinki University Medical School, Helsinki, Finland

**Keywords:** polymyxin B, colistin, extremely resistant (XDR), Gram-negative bacteria, improved polymyxins

In the original article, there was a mistake in Table 1 as published. The structure of SPR206 was partially incorrect. In the correct structure, R(FA) is (3*S*)-4-amino-3-(3-chlorophenyl)butanoyl, R1 is absent, and R3 is Dap^+^. The corrected Table 1 appears below.

**Table 1:** The structures of polymyxin B, colistin, and the novel polymyxin derivatives that display improved efficacy in animal infection models (compounds 4-10)^a^,^b^.

	**Compound**	**R (FA)**	**R1**	**R2**	**R3**	**R4**	**R5**	**R6**	**R7**	**R8**	**R9**	**R10**
1	Polymyxin B (PMB)	Methyloctanoyl/methylheptanoyl	-Dab^+^	-Thr	-Dab^+^	-cy[Dab	-Dab^+^	-DPhe	-Leu	-Dab^+^	-Dab^+^	-Thr]
2	Colistin (polymyxin E)	Methyloctanoyl/methylheptanoyl	-Dab^+^	-Thr	-Dab^+^	-cy[Dab	-Dab^+^	-DLeu	-Leu	-Dab^+^	-Dab^+^	-Thr]
3	CB-182,804	2-chloro-phenylamino-carbonyl	-Dab^+^	-Thr	-Dab^+^	-cy[Dab	-Dab^+^	-DPhe	-Leu	-Dab^+^	-Dab^+^	-Thr]
4	FADDI-002	Octanoyl	-Dab^+^	-Thr	-Dab^+^	-cy[Dab	-Dab^+^	-DPhe	-Ada	-Dab^+^	-Dab^+^	-Thr]
5	FADDI-287	Octanoyl	-Dab^+^	-Thr	-Dap^+^	-cy[Dab	-Dab^+^	-DLeu	-Abu	-Dab^+^	-Dab^+^	-Thr]
6	CA824	(S)-1-(2-methylpropyl)-piperazine-2-carbonyl^+^	–	-Thr	-Dab^+^	-cy[Dab	-Dab^+^	-DPhe	-Leu	-Dab^+^	-Dab^+^	-Thr]
7	SPR206	(3*S*)-4-amino-3-(3-chlorophenyl)butanoyl	–	-Thr	-Dap^+^	-cy[Dab	-Dab^+^	-DPhe	-Leu	-Dab^+^	-Dab^+^	-Thr]
8	MicuRx-12	3-(2,2-dimethyl-butanoyloxy)-propanoyl (ester bond)	-Dab^+^	-Thr	-Dab^+^	-cy[Dab	-Dab^+^	-DPhe	-Leu	-Dab^+^	-Dab^+^	-Thr]
9	NAB739	Octanoyl	–	-Thr	-DSer	-cy[Dab	-Dab^+^	-DPhe	-Leu	-Dab^+^	-Dab^+^	-Thr]
10	NAB815	Octanoyl	-Dab^+^	-Thr	-DThr	-cy[Dab	-Dab^+^	-DPhe	-Leu	-Abu	-Dab^+^	-Thr]
11	SPR741 (NAB741)	Acetyl	–	-Thr	-DSer	-cy[Dab	-Dab^+^	-DPhe	-Leu	-Dab^+^	-Dab^+^	-Thr]

*The structure of one discontinued derivative (compound 3) as well as that of the potentiator compound SPR741 (NAB741) are also shown*.

a *Amino acyl residues that differ from those in polymyxin B are boxed*.

b *Abu, aminobutyryl; Ada, aminodecanoyl; Dap, diaminopropionyl; cy, cyclic portion indicated with brackets; Dab, diaminobutyryl; FA, fatty acyl*.

The authors apologize for this error and state that this does not change the scientific conclusions of the article in any way. The original article has been updated.

